# Identification of a New Theca/Interstitial Cell-Specific Gene and Its Biological Role in Growth of Mouse Ovarian Follicles at the Gonadotropin-Independent Stage

**DOI:** 10.3389/fendo.2019.00553

**Published:** 2019-08-14

**Authors:** Masato Aoyama, Akira Shiraishi, Shin Matsubara, Kaoru Horie, Tomohiro Osugi, Tsuyoshi Kawada, Keiko Yasuda, Honoo Satake

**Affiliations:** ^1^Department of Chemistry, Biology, and Environmental Science, Faculty of Science, Nara Women's University, Nara, Japan; ^2^Bioorganic Research Institute, Suntory Foundation for Life Sciences, Kyoto, Japan

**Keywords:** theca cell, secondary follicle, follicle growth, transcriptome, Asporin/PLAP-1

## Abstract

Theca/interstitial cells are responsible for the growth and maturation of ovarian follicles. However, little is known about the theca/interstitial cell-specific genes and their functions. In this study, we explored transcriptomes of theca/interstitial cells by RNA-seq, and the novel biological roles of a theca cell marker, asporin (Aspn)/periodontal ligament-associated protein 1 (PLAP-1). RNA-seq detected 432 and 62 genes expressed specifically in theca/interstitial cells and granulosa cells isolated from 3-weeks old mouse ovaries. Gene ontology analysis demonstrated that these genes were largely categorized into four major groups: extracellular matrix organization-related terms, chemotaxis-related terms, the angiogenesis-related terms, and morphogenesis-related terms. *In situ* hybridization demonstrated that the newly detected representative gene, *Aspn/PLAP-1*, was detected specifically in the outer layer of theca cells in contrast with the expression of the basal lamina-specific gene, Nidgen-1. Intriguingly, an Aspn/PLAP-1 antibody completely arrested the growth of secondary follicles that is the gonadotropin-independent follicle developmental stage. Furthermore, transforming growth factor-β (TGF-β)-triggered signaling was induced by the Aspn/PLAP-1 antibody treatment, which is consistent with the inhibitory effect of Aspn/PLAP-1 on TGF-β. Altogether, these results suggest that theca cells are classified into subpopulations on the basis of new marker genes and their biological functions, and provide evidence that Aspn/PLAP-1 is expressed exclusively in the outer layer of theca cells and plays a pivotal role in the growth of secondary follicles via downregulation of the canonical TGF-β signaling cascade.

## Introduction

In dioecious species, generation of fertile oocytes is an essential biological process. Oocytes are grown and matured in follicles, and ovulated from the ovary. Follicle development is a multi-step and complexed event controlled by a wide variety of endogenous factors. In mammals, a follicle is composed of a single oocyte, granulosa cells, and theca cells, and follicle development is largely classified into two stages: gonadotropin-dependent and independent stages. The former, namely, the antral follicle, is regulated by the hypothalamus-pituitary-gonad (HPG) axis via secretion of gonadotropins, LH and FSH, to the ovary in an endocrine fashion only at sexually mature individuals. In response to LH, theca cells produce androgen that is converted into estrogen by specific aromatases in response to FSH in granulosa cells, indicating that a major biological role of theca cells is production of sexual steroid hormones at the antral follicles (1–3). The latter is initiated at the fetus, where primordial follicles are generated. After birth, the primary, secondary and preantral follicles grow sequentially via the regulation exerted by diverse endogenous factors in the intraovarian paracrine/endocrine networks, in a manner independent of gonadotropins (1–5). In secondary follicles, theca cells are differentiated from interstitial (stromal) cells, and start the formation of the thecal layers and the basal lamina, indicating that theca cells are responsible for maintenance of physical structure of follicles (1–7). Moreover, interstitial cells, differentiating into theca cells, were found to be necessary for the normal growth of mouse secondary follicles *in vitro* (5, 8). However, the net molecular mechanisms of follicle growth at gonadotropin-independent stages remain largely unknown.

To date, various endogenous secretory or membrane-bound ligands and their receptors, including transforming growth factor (TGF)-β, have been shown to be expressed in theca/interstitial cells, supporting the notion that theca/interstitial cells both undergo and induce extensive cellular signaling (1–3, 9–11). Over the past 5 years, transcriptomes for theca/interstitial cells have been increasingly documented, leading to the detection of several theca/interstitial cell-specific genes (11–24). These findings suggest more multiple pivotal roles of theca/interstitial cells in the follicle development than what is known, in particular, at gonadotropin-independent development stages. Nevertheless, most studies have investigated the effect of these factors on isolated theca/interstitial cells, not on isolated follicles (1–5, 7). Furthermore, transcriptomes have been restricted to theca/interstitial cells of antral follicles, and the localization of the gene expression detected by transcriptomic analysis has not fully been observed in the ovary or follicles (11–24). Such shortcomings hinder the elucidation of biological roles of theca/interstitial cells in the gonadotropin-independent follicle growth and the underlying molecular mechanisms. In the present study, we investigated the expression of new theca/interstitial cell-specific genes, and show novel regulation of secondary follicle growth by asporin (Aspn)/periodontal ligament-associated protein 1 (PLAP-1), an endogenous negative factor for TGF-β signaling cascades.

## Materials and Methods

### Animals

Female ICR mice (Japan SLC, Inc., Shizuoka, Japan) were maintained under controlled light conditions (14L:10D) and were given food and water *ad libitum*. The day of birth was designated as Day 0. On Day 1, each mother was left with eight pups to equalize the growth of pups between litters. Our investigations were conducted in accordance with the Animal Care Committee of Nara Women's University.

### Preparation of Follicles, Theca/interstitial Cells, and Granulosa Cells

Secondary follicles, theca/interstitial cells, and granulosa cells were collected from the ovaries of 20 3-weeks old different mice as previously described (6).

### RNA-Seq

Total RNA was extracted from ovarian interstitial cells and granulosa cells using RNeasy Mini Kit (Qiagen Gmbh., Hilden, Germany). The quality of the RNA samples was evaluated by using BioAnalyzer (Agilent Technologies, CA, USA) with RNA6000 Nano Chip. A 1-μg aliquot of total RNA from each sample was used to construct cDNA libraries using TruSeq Stranded mRNA Sample preparation kit (Illumina, CA, USA), according to the manufacturer's instructions. The resulting cDNA library was validated using BioAnalyzer with DNA1000 Chip and quantified using Cycleave PCR Quantification Kit (TAKARA BIO INC., Shiga, Japan). Single end sequencing using 101 cycles was performed using HiSeq1500 (Illumina) in the rapid mode. Total reads were extracted with CASAVA v1.8.2 (Illumina). The resulting fastq files were deposited in Sequence Read Archive (SRA) under accession numbers SRR5097519 (Theca/Interstitial Cell, rep. 1), SRR5097520 (Theca/Interstitial Cell, rep. 2), SRR5097517 (Granulosa cells, rep. 1), and SRR5097518 (Granulosa cells, rep. 2) (Table 1). Then, PCR duplicates, adaptor sequences and low-quality reads were removed from the extracted reads as follows. Briefly, if the first 10 bases of the two reads were identical and the entire reads exhibited >90% similarity, the reads were considered PCR duplicates. Remaining reads were then aligned with Bowtie version 2.2.3 to the mouse transcriptome mm10 with refseq ID, which was downloaded from ENSEMBL database. The difference of expression levels was calculated with edgeR (25), which is the Bioconductor package based on negative binomial generalized linear models.

**Table 1:** The numbers of resultant reads and mapping rate.

**Samples**	**Theca/Interstitial cell, rep. 1**	**Granulosa cell, rep. 1**	**Theca/Interstitial cell, rep. 2**	**Granulosa cell, rep. 2**
SRA ID	SRR5097519	SRR5097518	SRR5097520	SRR5097517
Reads	26,752,459	26,226,341	31,233,081	32,256,405
Mapped reads	22,125,009	21,688,635	24,166,670	26,159,002
Mapping rate	82.7%	82.7%	77.4%	81.1%

### GO Enrichment Analysis

To predict the function of ovarian interstitial cells and granulosa cells -specific transcripts, the sequences with lower *p*-values than 0.025 were analyzed using GO enrichment analysis, as described previously (26). The GO terms for the transcripts were downloaded from Mouse Genome Informatics database (MGI). Briefly, to quantify the enrichment of GO terms, we calculated enrichment scores as follows:

$$\begin{array}{l} Enrich\;\left(GO\right)=\log_{2}\frac{{\;}^{N_{Gr<<In}\left(GO\right)}\text{​}{╱}_{N_{Total}\left(Gr<<In\right)}\;}{{\;}^{N_{all}\left(GO\right)}\text{​}{╱}_{N_{Total}\left(all\right)}\;} \end{array}$$

where Nx(GO) indicates the frequency of each GO term for theca/interstitial cell (Gr < < In)-specific or whole transcriptome genes (i.e., x), and Ntotal(X) indicates the frequency of theca/interstitial cell (Gr < < In)-specific or whole mouse transcriptome genes (i.e., X) mapped to each GO term in MGI database. For correction, a pseudo-count was set as fixed value (0.05).

### Real-Time PCR

Theca/interstitial cells and granulosa cells were isolated from the ovaries of 30 individuals of 3-weeks-old mice as previously described (5). Total RNA was extracted from cells or follicles using RNeasy Plus Mini kit (Qiagen Gmbh), and reverse-transcribed to the template cDNA at 50°C for 50 min using the oligo dT anchor primer and SuperScript™ III Reverse Transcriptase (Thermo Fisher Scientific). The real-time PCR was performed using CFX96 Real-time System and SsoAdvanced™ Universal SYBR Green Supermix (Bio-Rad laboratories, Hercules, CA, USA). Total volume of reaction mixtures was 20 μl, consisting of 100 ng template cDNA, each 500-nM primers and 10 μl SYBR Green Master Mix solution. The real-time PCR was performed for initial steps at 95°C for 30 s, followed by 44 cycles at 95°C for 15 s, and at 60°C for 30 s. The melting curve analysis was performed to confirm the absence of primer dimers. In brief, ΔCt values for the β-actin gene expression were used as standard values, and 2^−ΔΔCt^ values for theca/interstitial cells-high level expressed genes were calculated according to the manufacturer's instruction. Primers were designed using Primer-blast web tool (NCBI). Sequences of the primers used for the real-time PCR are listed in Supplemental Data 1.

### *In situ* Hybridization in the Mouse Ovary

The open reading frames of *Nid1* (nt 1,246–1,643) and *Aspn/Plap-1* (nt 267–668) were amplified using ovary cDNA and gene-specific primers (Supplemental Data 2), respectively. The PCR program was 94°C for 3 min, 35 cycles of 94°C for 30 s, 50°C for 30 s, and 72°C for 45 s, and final extension at 72°C for 7 min. Each of the PCR products was inserted into the pCRII-TOPO dual promoter vector (Thermo Fisher Scientific) according to the manufacturer's instruction and supplied to preparation of RNA probes as a template. Digoxigenin-labeled RNA antisense and sense probe for each gene was prepared using a digoxigenin-labeled RNA labeling kit (Roche Diagnostics, Switzerland). Ovaries of 3-weeks female mice were dissected and fixed in 4% paraformaldehyde in PBS at 4°C overnight. The fixed tissues were soaked in a refrigerated sucrose solution (10% in PBS) for 30 min at room temperature. The tissues were then soaked in a 15% sucrose solution at 4°C overnight until they sank. The ovaries were embedded in Super Cryoembedding Medium-L1 (Leica Microsystems Japan, Tokyo, Japan) and sectioned at a 10 μm thickness with a CryoStar NX70 cryostat (Thermo Fisher Scientific Inc.) at −18°C. The sections were placed onto FRONTIER-coated slides (FRC-04; Matsunami Glass Ind., Ltd., Osaka, Japan). Hybridization, washing and detection were carried out as previously reported (27–29). No positive signals were observed when sense probes were used, confirming the specificity of hybridization.

### Morphological Change of Secondary Follicles by an Endogenous Aspn/PLAP-1 Inhibition

Co-cultivation of follicles with theca/interstitial cells using collagen gel (Cellmatrix Type I-A; Nitta Gelatin, Inc., Japan) was performed as previously described (5, 6). In brief, 0.2% collagen gel containing 10% FBS (Thermo Fisher Scientific Inc.), 100 U/ml penicillin, 0.1 mg/ml streptomycin (Nacalai Tesque Inc., Kyoto, Japan) and Dulbecco's Modified Eagle Medium components (Nissui Pharmaceutical Co., Ltd., Tokyo, Japan) was used to culture the secondary follicles. Approximately 20–30 secondary follicles with 100 μm-diameter and 6 × 10^4^/well-theca/interstitial cells were co-cultured with the collagen gel only, and the collagen gel containing 10 μg/ml-normal rabbit IgG (FUJIFILM Wako Pure Chemical Corporation, Osaka, Japan) or 10 μg/ml of anti-Aspn/PLAP-1 rabbit polyclonal antibody (Cloud-Clone Corp, TX, USA) at 37°C for 5 days in a 96-well plate. Morphological change of each follicle was observed using an inverted microscopy Olympus CK2 (Olympus, Tokyo, Japan).

### Western Blotting of Smad Proteins in Aspn/PLAP-1 Antibody-Treated Ovaries

Ovaries were isolated from 3-weeks old mice, and cut longitudinally into symmetrical half-pieces. Each half-portion of the mouse ovaries was incubated with the culture medium containing anti-Aspn/PLAP-1 antibody, normal rabbit IgG (Wako, Japan), TGF-β1 (R&D systems, MN, USA), or BMP2 (R&D systems) at 37°C for 0–3 h. The cultured ovaries were homogenized in 100 μl of RIPA buffer (Thermo Fisher Scientific Inc.) containing cOmplete, EDTA-free (Roche) and PhosSTOP (Roche) at 4°C for 1–2 min at 28 Hz using Tissue lyser II (Qiagen Gmbh). The homogenate was incubated on ice for 10 min, followed by centrifugation at 15,300 g for 10 min at 4°C. The protein amount in the supernatant was measured using BCA protein assay kit (Thermo Fisher Scientific Inc.). 40 μg of the resultant ovarian protein lysate was separated on 5–20% gradient SDS-PAGE gel, and was electroblotted onto PVDF membranes. The membranes were blocked by Block-Ace (DS pharma biomedical, Osaka, Japan) for 1 h at room temperature, and were treated with a 1:1,000–2,000 dilution of an antibody against Smad2/3 (SantaCruz Biotechnology, CA, USA), phospho-Smad2/3 (p-Smad2/3) [Cell Signaling Technology (CST), MA, USA] Smad1/5/9 (Abcam, Tokyo, Japan) or p-Smad1/5/9 (CST) in the Can get signal 1 solution (TOYOBO, Osaka, Japan) overnight at 4°C. After washing with 0.05% Tween TBS (TBST), the membranes were incubated with a 1:2,000 HRP-conjugated donkey anti-rabbit secondary antibody (GE healthcare, IL, USA) in Can get signal 2 solution (TOYOBO) for 1 h at room temperature. The anti-GAPDH antibody (Abcam) and the corresponding secondary antibody (GE healthcare) in TBST were used as an internal control. Signals were detected using a chemiluminescent detection system, ECL Select Detection Reagent according to the manufacturer's instruction (GE healthcare).

### Statistical Analysis

Results are shown as mean ± SE. Data were analyzed by one-way ANOVA with Turkey's multiple comparison tests. Differences were accepted as significant for *P* < 0.05.

## Results

### Detection of Mouse Theca/Interstitial Cell-Specific Genes

**Figure 1:**
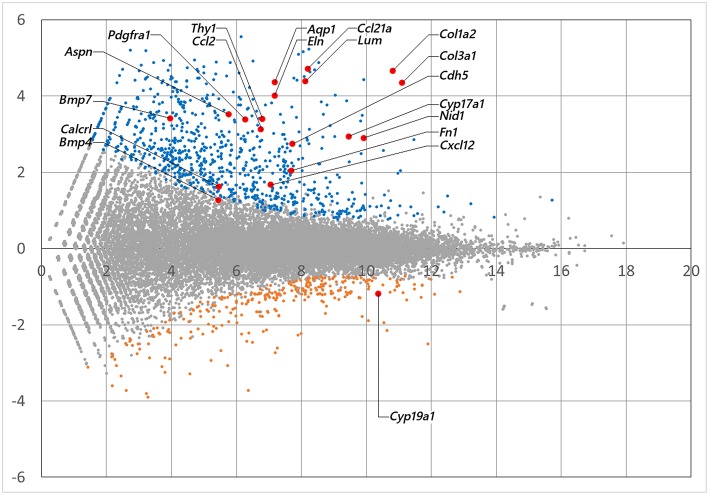
The M-A plot showing the distribution of each gene expression. A and M stands for basemean of normalized expression data and log (fold change) for each gene in theca/interstitial and granulosa cells, respectively. Blue, orange, and red dots indicate theca-upregulated genes (*P*_adj_ < 10^−6^), granulosa-upregulated genes (*P*_adj_ < 10^−6^) and genes validated in real-time RT-PCR (Figure 3), respectively.

mRNA library of mouse ovarian theca/interstitial cells and that of granulosa cells were sequenced using Hiseq 1500, and 26–32 million reads for 101 single end reads were obtained from two replicates for each samples (Table 1). After mapping to the mouse transcriptome, 77–82% of the reads were mapped, displaying 21,028 and 20,425 expressed genes with more than 10 reads on granulosa cell and theca/interstitial cell, respectively. Subsequently, we compared the gene expression levels between theca/interstitial cell and granulosa cell using EdgeR pipeline, detecting 432 and 62 upregulated genes with lower *p*-values than 10^−6^ in theca/interstitial cell and granulosa cell, respectively (Figure 1).

*CYP17A1, BMP-4*, and *-7* genes, known theca/interstitial cell markers were shown to be intensely expressed in theca/interstitial cells (Table 2). These data are consistent with the previous findings (1–3, 11, 30), confirming the detection of differentially expressed genes by the RNA-seq method.

**Table 2:** Differential expression of marker genes for theca/interstitial cells per granulosa cells.

**Genbank ID**	**Gene symbol**	**Fold-change**	***p*-value**
NM_007809	Cyp17a1	7.68	9.41 × 10^−36^
NM_007554	Bmp4	2.41	4.41 × 10^−3^
NM_007557	Bmp7	10.49	2.68 × 10^−12^

**Figure 2:**
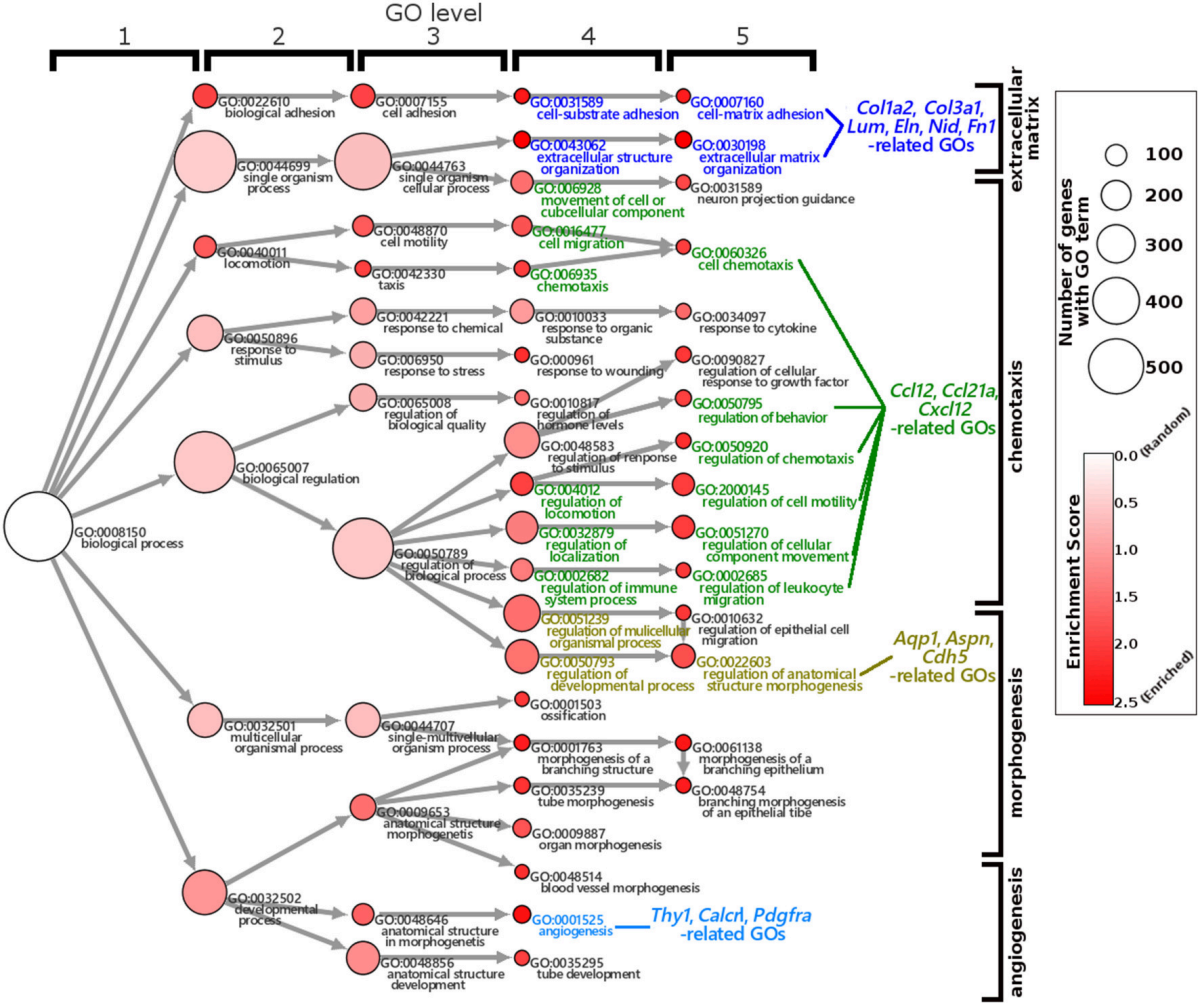
Gene Ontology enrichment analysis of differentially expressed genes in theca/interstitial cells and granulosa cell. Each node represents a GO term. The branches of GO hierarchical trees without significantly enriched GO terms are not shown. The circle size of each node indicate the number of genes with the GO terms. The colors of each node represent the enrichment score. Red indicates high enrichment score of expression genes in theca/interstitial cells. Edges represent “is_a” connections between GO terms. Blue, green, yellow, and light blue GO terms indicate the GO terms related to the genes validated in real-time RT-PCR (Figure 3), respectively.

The GO enrichment analysis against upregulated genes in each cell detected 35 and 9 GO terms with GO-levels at 4–5 as enriched GO terms with more than 2 in GO enrichment score in granulosa cell and theca/interstitial cell, respectively (Table 3). As depicted in Figure 2, 39 theca/interstitial cell-enriched GO terms were grouped to four groups; extracellular matrix organization-related terms, chemotaxis-related terms, the angiogenesis-related terms and morphogenesis-related terms. For the extracellular matrix organization-related terms, extracellular structure organization (GO:0043062), extracellular matrix organization (GO:0030198), cell substrate adhesion (GO:0031589) and cell-matrix adhesion (GO:0007160), which are involved in extracellular matrix organization, were enriched in theca/interstitial cell. For example, collagen type I alpha 2 (*Col1a2*), collagen type III alpha 1 (*Col3a1*), lumican (*Lum*), elastin (*Eln*), nidogen 1 (*Nid1*), and fibronectin 1 (*Fn1*) were shown to be 25.31-, 20.37-, 20.96-, 16.04-, 7.46-, and 4.12-fold expressed in theca/interstitial cell, respectively, compared with granulosa cells (Figures 1, 2, and Table 3). These results are in good agreement with the previous studies demonstrating that theca/interstitial cells participate in the follicle development via production of various extracellular matrix and cell adhesion proteins (1–3, 6, 31–33). Particularly, the high expression of Fn1 and Nid1, which are basal lamina-specific adhesive molecules in extracellular matrix (1, 2, 30, 31), suggests the biological roles for the theca/interstitial cells in production of basal membranes of follicles.

**Table 3:** Upregulated genes in theca/interstitial cells.

**Genbank ID**	**Gene symbol**	**Fold-change**	***p*-value**	**Related GOs**
NM_007743	*Col1a2*	25.31	1.34 × 10^−81^	GO:0043062, GO:0030198, GO:0031589, GO:0007160
NM_009930	*Col3a1*	20.37	4.43 × 10^−70^	
NM_008524	*Lum*	20.96	3.51 × 10^−58^	
NM_007925	*Eln*	16.04	1.28 × 10^−42^	
NM_010917	*Nid1*	7.46	1.27 × 10^−30^	
NM_010233	*Fn1*	4.12	1.51 × 10^−10^	
NM_011333	*Ccl2*	8.75	5.99 × 10^−23^	GO:0006928, GO:0016477, GO:0060326, GO:006935, GO:0050795, GO:0050920, GO:2000145, GO:0040012, GO:0051270, GO:0032879, GO:002685
NM_011124	*Ccl21a*	20.37	9.22 × 10^−74^	
NM_021704	*Cxcl12*	3.20	6.84 × 10^−8^	
NM_009382	*Thy1*	10.60	3.76 × 10^−33^	GO:0001525
NM_018782	*Calcrl*	3.08	7.56 × 10^−7^	
NM_011058	*Pdgfra*	10.44	1.26 × 10^−24^	
NM_007472	*Aqp1*	20.48	9.16 × 10^−48^	GO:0022603, GO:0050793, GO:0051239
NM_025711	*Aspn/Plap-1*	11.46	1.98 × 10^−10^	
NM_009868	*Cdh5*	6.70	4.58 × 10^−14^	

Chemoattractants *Ccl2, Ccl21a*, and *Cxcl12* were 8.75-, 20.37-, and 3.20-fold upregulated in theca/interstitial cell, respectively (Figures 1, 2, and Table 3) which are contained in various GO terms related to chemotaxis; for instance, movement of cell or subcellular component (GO:0006928); cell chemotaxis (GO:0060326).

The GO term angiogenesis (GO:0001525), including cell antigen 1 theta (*Thy1*), calcitonin receptor-like (*Calcrl*), platelet derived growth factor receptor alpha (*Pdgfra*), was also enriched in theca/interstitial cell. *Thy1, Calcrl*, and *Pdgfra* were 10.60-, 3.08-, and 10.44-fold upregulated in theca/interstitial cell, respectively, compared with granulosa cells (Figures 1, 2, and Table 3). These angiogenesis-related genes coincide with the vascularity of interstitial cell and theca/interstitial cell (1, 2, 6, 34).

**Figure 3:**
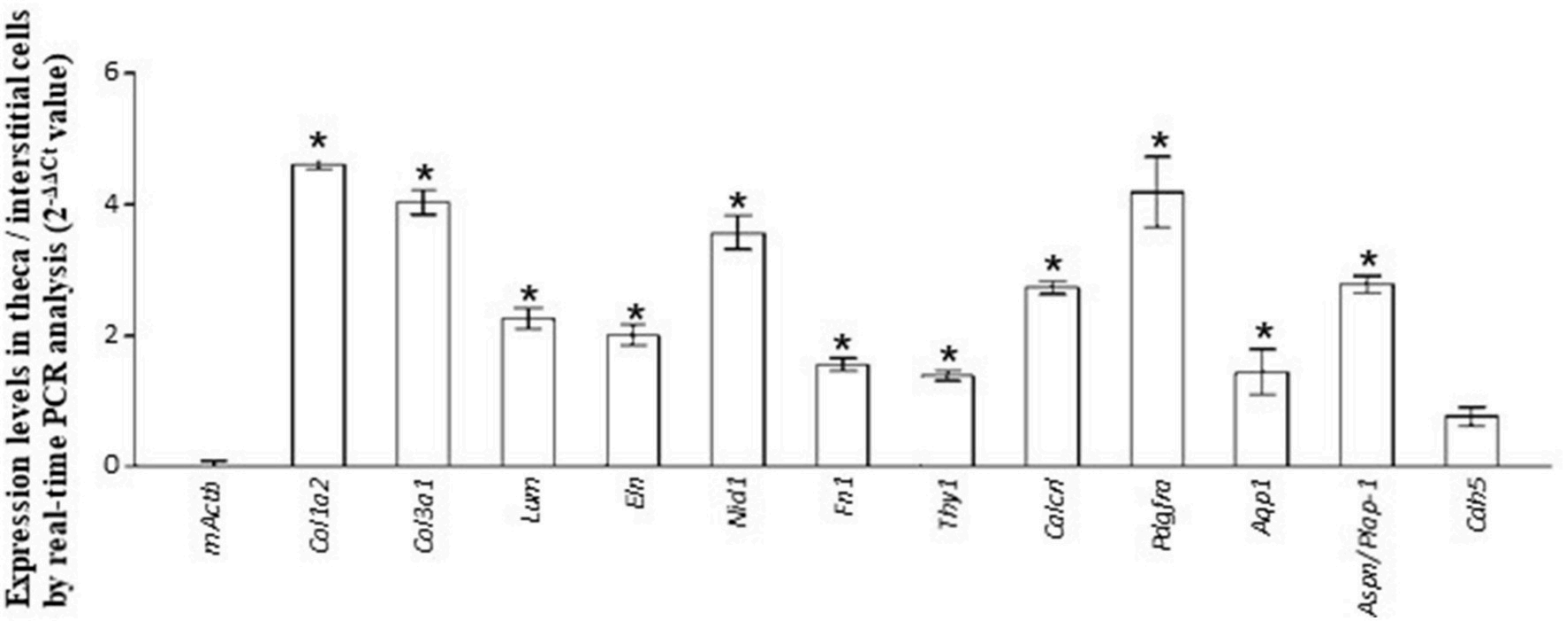
Validation of RNA-seq. data by real-time PCR. The expression level of each gene in theca/interstitial cells is calculated using the 2^−ΔΔCt^ values. Data are shown as the means of three independent experiments ± SE. Data were analyzed by a combination of one-way ANOVA and turkey's multiple comparison tests. *P < 0.05 vs. mouse actin β (mActb).

The GO terms related to morphogenesis, including regulation of anatomical structure morphogenesis (GO:0022603); regulation of developmental process (GO:0050793); and regulation of multicellular organismal process (GO:0051239), were enriched in theca/interstitial cell. Aquaporin 1 (*Aqp1*), *Aspn/PLAP-1*, and Cadherin 5 (*Cdh5*), which were categorized into all of these GO terms, were 20.48-, 11.46-, and 6.70-fold upregulated in theca/interstitial cell, respectively (Figures 1, 2, and Table 3), suggesting the role of theca/interstitial cells in some developmental processes. Furthermore, the expression of aforementioned 12 genes in theca/interstitial cells were examined by real-time PCR. As shown in Figure 3, real-time PCR demonstrated that the 11 genes were more intensely expressed in theca cells/interstitial cells than granulosa cells, validating the RNA-Seq data.

### Localization of *Nid1* and *Aspn* in the Mouse Ovary

**Figure 4:**
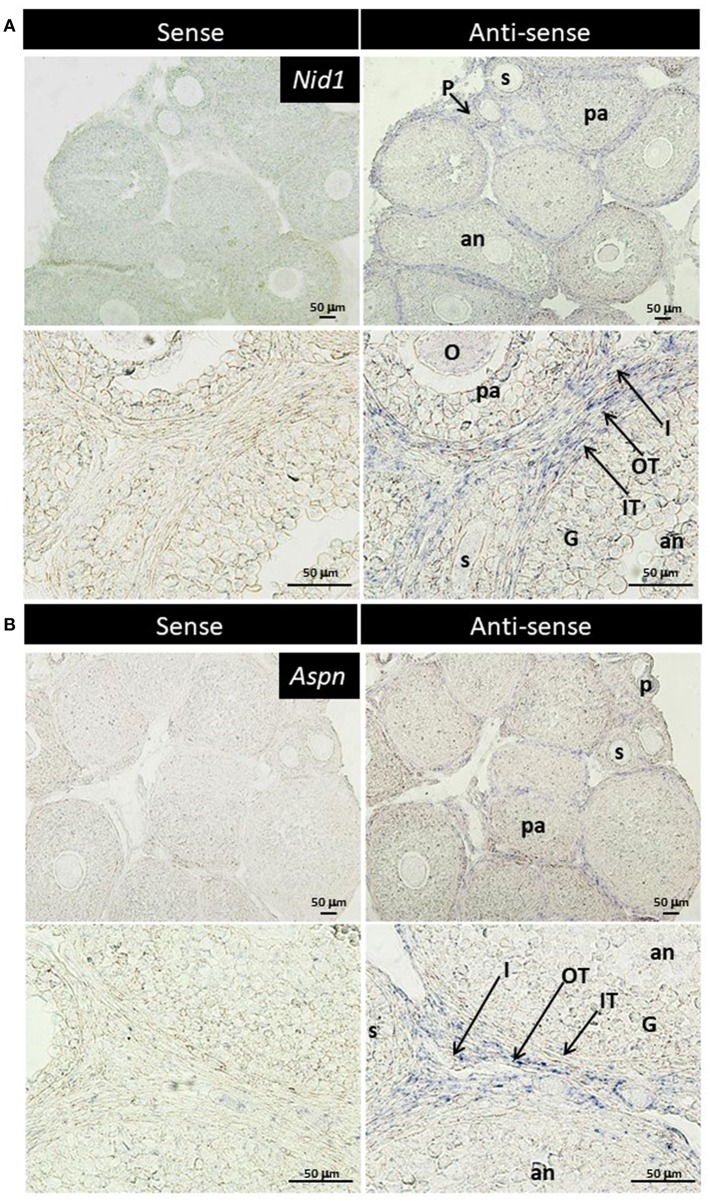
Localization of nidgen1 (*Nid1)* mRNA **(A)** and Aspn/PLAP-1 (*Aspn)* mRNA **(B)** in the 3-weeks-old mouse ovary is shown. *Nid1* was localized to the theca cell layer in the follicles later than secondary stage (right panels in **A**). These genes were also expressed in the interstitial cells. *Aspn* was expressed in the interstitial cells and the outer layer of theca cells but not in the inner layer of theca cells (right panels of **B**). No specific signal was found with any of the sense probes (left panels). O, oocytes; G, granulosa cell, IT, the inner layer of theca cells; OT, the outer layer of theca cells; I, interstitial cell; P, primary follicle; s, secondary follicle; pa, preantral follicle; an, antral follicle. Scale bars, 50 μm.

TGF-β is found to exhibit some biological effects on isolated theca cells and granulosa cells (35, 36), and Aspn/PLAP-1, a small leucine-rich repeat protein, plays endogenous inhibitory roles for the TGF-β signaling (37–42). These findings suggest some biological roles of Aspn/PLAP via regulation of TGF-β signaling in the ovary. However, localization of their mRNAs has yet to be examined. Subsequently, we thus performed *in situ* hybridization of *Nid1* (as a reference to typical theca-specific localization) and *Aspn/Plap-1* in the ovary. *Nid1* was shown to be expressed in theca cells and interstitial cells of secondary, preantral and antral follicles but not in granulosa cells or oocytes (Figure 4A). Interestingly, expression of *Aspn/Plap-1* was detected specifically in the outer layer of theca cells and the adjacent interstitial cells but not in the inner layer of theca cells of secondary, preantral, and antral follicles (Figure 4B). Altogether, these results provide evidence for the specific and differential expression of *Nid1* and *Aspn*/*Plap-1* in theca/interstitial cells in the ovary.

### Biological Roles for *Aspn/PLAP-1* in the Follicle Growth

The unique localization of the *Aspn/Plap1* gene expression in the outer layer of theca cells (Figure 4B) suggested the involvement of Aspn/PLAP1 in the formation of the theca cell layers and the follicle growth. We thus evaluated the effect of Aspn/PLAP-1 on the growth of secondary follicles in a collagen gel medium (4). Aspn/PLAP-1 was formerly shown to suppress a TGF-β-Smad signaling cascade via interaction with TGF-β (11, 37–42).

**Figure 5:**
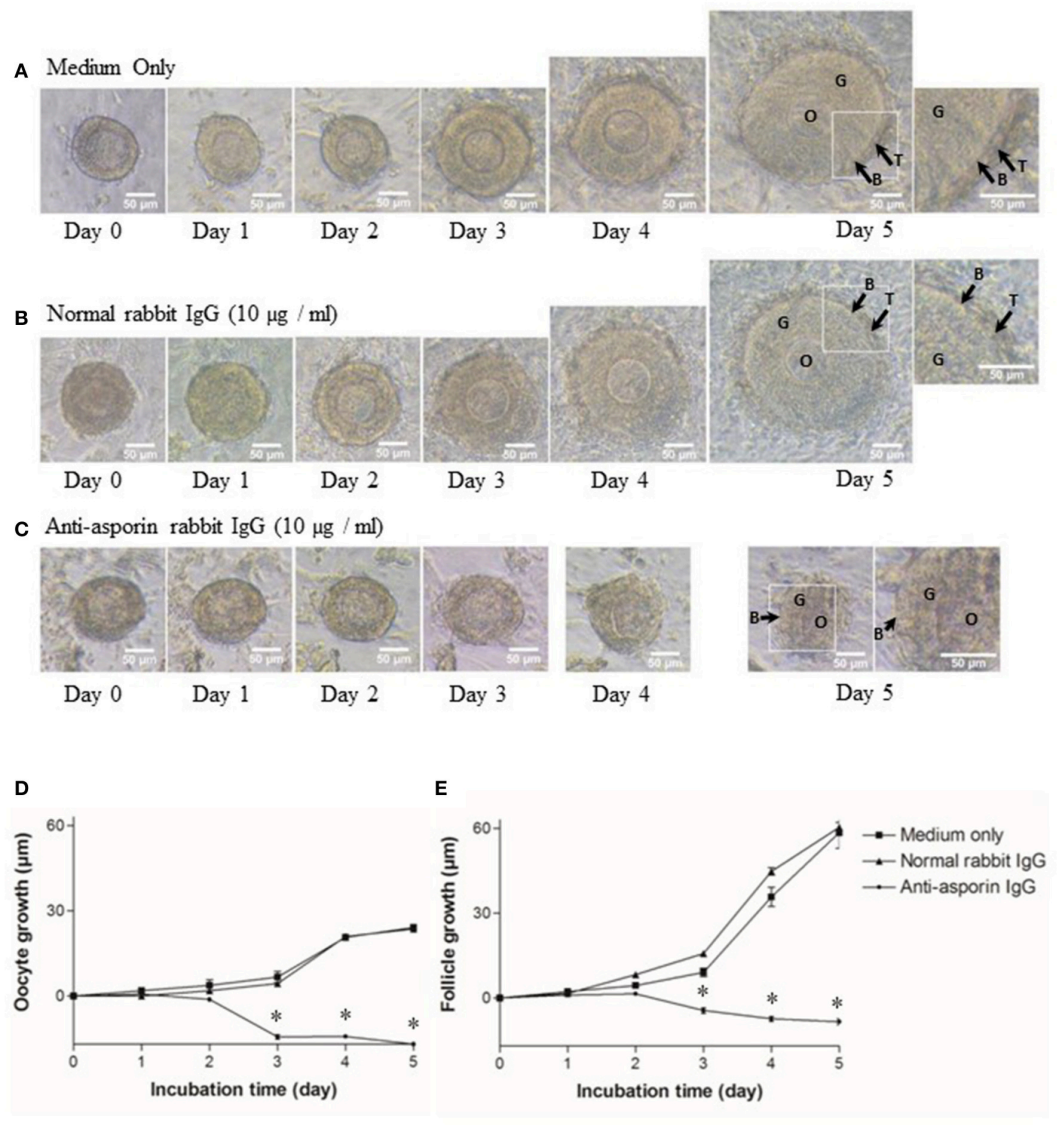
Morphological change and growth inhibition of secondary follicles by an endogenous asporin/PLAP-1 inhibition. Secondary follicles were incubated for days with no antibody **(A)**, 10 μg/ml normal rabbit IgG **(B)**, or 10 μg/ml anti-asporin IgG **(C)**. Treatment with 10 μg/ml anti-asporin IgG resulted in complete loss of the growth of all secondary follicles and of the theca cell layer formation (**C** and closed circle of **D,E**). Moreover, ~70% of the non-grown secondary follicles were shrunk in the presence of the anti-asporin IgG **(C)**. Three independent experiments using 20–30 follicles were performed. O, oocyte; G, granulosa cell; B, basal lamina, T, theca cells. Scale bars, 50 μm. Size change in the diameter of oocytes **(D)** and follicles **(E)** of the secondary follicles treated with the medium only (solid square), 10 μg/ml normal rabbit IgG (solid triangle), or 10 μg/ml anti-Aspn/PLAP-1 rabbit IgG (solid circle). The means ± SE are obtained from three independent experiments using 20–30 follicles. Data were analyzed by a combination of one-way ANOVA and turkey's multiple comparison tests. *P < 0.05 vs. medium only group and normal rabbit IgG group.

As previously reported (5, 6), all secondary follicles grew to preantral follicles, and interstitial cells developed to theca cell layers in 5 days (Figures 5A,D,E). Hence, we examined whether Aspn/PLAP1 affected the growth of secondary follicles in the presence of an anti-Aspn/PLAP-1 antibody. A striking feature is that treatment of secondary follicles with the anti-Aspn/PLAP-1 antibody resulted in complete loss of the growth of all secondary oocytes and follicles and of the theca cell layer formation (Figures 5C–E), whereas normal IgG is devoid of any effects (Figures 5B,D,E). Moreover, ~70% of the non-grown secondary oocytes and follicles displayed shrunk shape in the presence of the anti-Aspn/PLAP-1 antibody (Figures 5C–E), but the basal lamina remains (Figure 5C). Altogether, these results revealed that Aspn/PLAP-1 plays a crucial role in the growth of secondary follicles.

**Figure 6:**
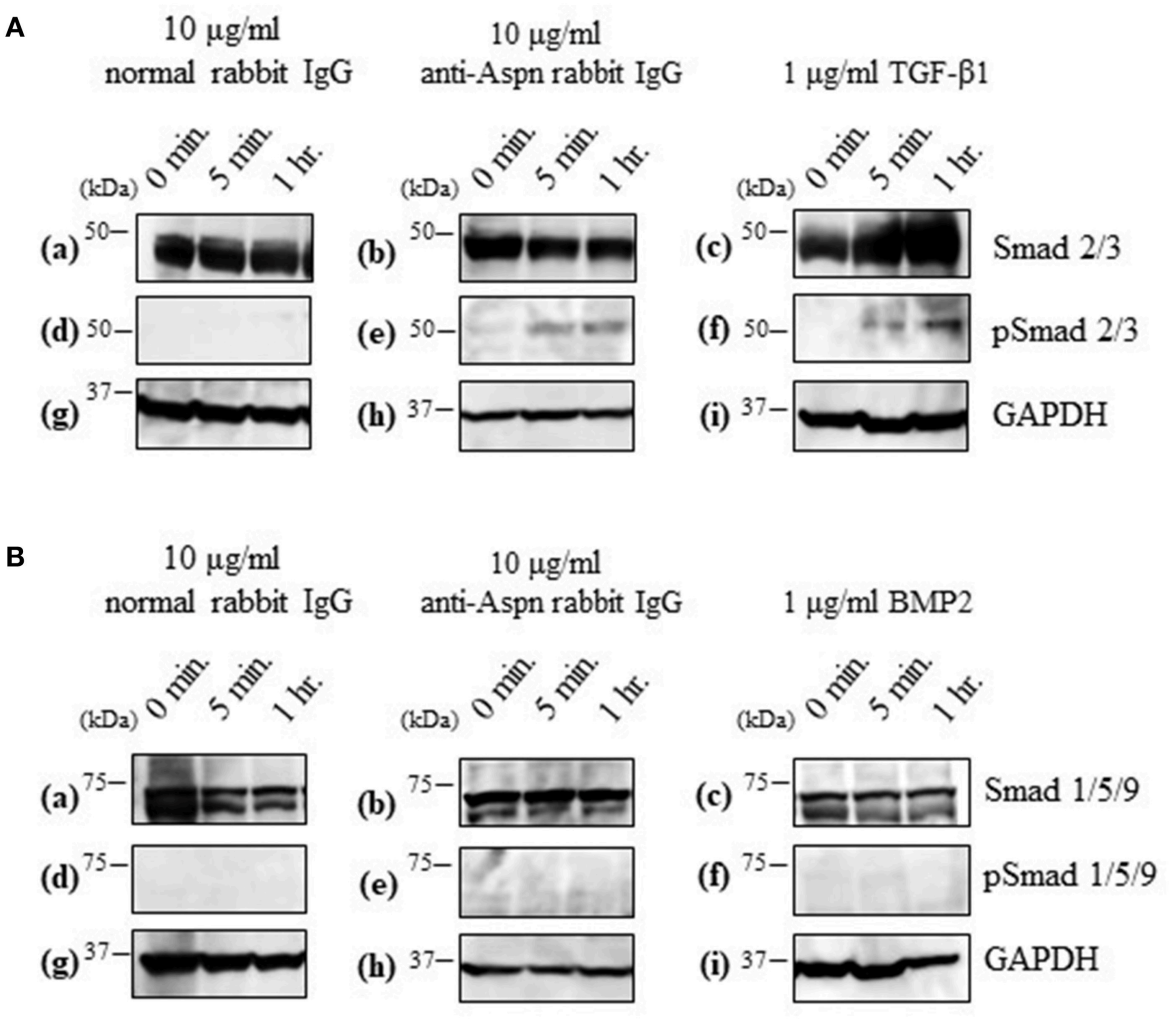
Western blotting of inducible phosphorylated Smad proteins in ovaries treated with 10 μg/ml normal antibody [left panel; (a,d,g)], 10 μg/ml anti-Aspn/PLAP-1 antibody [middle panel; (b,e,h)], 1 μg/ml TGF-β1 or 1 μg/ml BMP2 [right panel; (c,f,i)]. **(A)**, Phosphorylation of Smad2/3 was detected in the presence of anti-Aspn/PLAP-1 antibody or TGF-β1. **(B)**, Phosphorylation of Smad1/5/9 was not detected in the anti-Aspn/PLAP-1 antibody- or BMP2-treated ovary. No Smad phosphorylation was observed in the ovary treated normal rabbit IgG. GAPDH expression was used as the protein loading control.

Aspn/PLAP-1 has been shown to bind to TGF-β and BMP2, and suppress their functions (37–42). Combined with the aforementioned inhibition of the secondary follicle growth by the anti-Aspn/PLAP-1 antibody (Figure 5), the present data suggest that the TGF-β-Smad2/3 signaling pathways is involved in the regulation of the secondary follicle growth. We thus examined the phosphorylation of Smad2/3 in the ovaries in the presence or absence of the anti-Aspn/PLAP1 antibody, given that the phosphorylation of Smad2/3 and Smad1/5/9 are induced by TGF-β and BMP2, respectively (35, 36, 43–45). No significant signals of phosphorylation of Smad proteins were detected in normal rabbit IgG-treated ovaries (Figures 6A-d,B-d). Notably, treatment of the ovaries with the anti-Aspn/PLAP-1 antibody resulted in phosphorylation of Smad2/3 for 5-min and 1-h incubation (Figure 6A-e), which is typical rapid and persistent TGF-β-specific Smad phosphorylation pattern (35, 36, 43–45), whereas phosphorylation of Smad1/5/9 was not observed (Figure 6B-e). Likewise, TGF-β1 induced phosphorylation of Smad2/3 in the ovary (Figure 6A-f), but BMP2 failed to phosphorylate Smad1/5/9 (Figure 6B-f). Altogether, these results proved that Aspn/PLAP-1 regulates normal growth of the secondary follicles via suppression of the canonical TGF-β-Smad2/3 signaling pathway.

## Discussion

Theca/interstitial cells are believed to participate in the follicle development and maturation via a wide range of endocrine/paracrine pathways. However, the biological significance of theca/interstitial cells in the growth of follicles largely remains unclear. In particular, these cells are expected to play crucial roles in the growth of secondary and preantral follicles as well as antral and preovulatory ones, given that such early-stage follicles are not regulated by gonadotropins from the HPG axis. Thus, a series of identification of theca/interstitial cell-specific marker genes and elucidation of their localization in follicles and their intra-follicular functions are expected to contribute to the verification of the regulatory mechanisms underlying the development of follicles by theca/interstitial cells at gonadotropin-independent stages.

RNA-seq using a next-generation sequencer is a powerful tool for the identification of gene expression profiles and their dynamics in target tissues. To date, gene expression profiles of theca/interstitial cells have been investigated in several mammals including bovine, equine, rodents, and primates (13–24, 45). Nevertheless, little is known about the gene expression of theca/interstitial cells in secondary and preantral follicles namely, gonadotropin-independent stage-follicles, given that most of transcriptomes are limited to theca/interstitial cells in antral and preovulatory follicles (13–24, 45). Furthermore, localization of the genes detected by transcriptomes has poorly been investigated. In this study, we have explored transcriptomes and the localization in theca/interstitial cells in secondary to antral follicles in sexually immature mice where gonadotropins do not function yet.

RNA-seq and real-time PCR revealed the specific expression of genes encoding component proteins of extracellular matrix and basal lamina adhesive molecules, including Col3a1 and Nid1, in theca/interstitial cells (Figures 1–3). These expression profiles are compatible with the previous studies (2, 3, 6, 17–21, 32, 33) showing that some theca cells are responsible for construction of the basal lamina and extracellular matrix in various mammalian follicles. Gene expression of a typical basal lamina component, Nid1, was detected in multiple, but not all, the inner and outer layers of theca cells in secondary, preantral, and antral follicles (Figure 4A). These results indicated that some populations of theca cells participate in formation of the basal lamina.

Various signaling molecule-related genes were also shown to be expressed specifically in theca/interstitial cells: including Aspn/PLAP-1 (Figures 1–3). To our knowledge, this is the first report showing the expression of Aspn/PLAP-1 in theca/interstitial cells. Furthermore, the gene expression of Aspn/PLAP-1 was shown to be limited to the outer layer of the theca of the secondary, preantral, and antral follicles (Figure 4B). Such unique and unprecedented localization led to the identification of the Aspn/PLAP-1 gene as a novel specific marker for the thecal outer layer.

Of particular significance is that neutralization of Aspn/PLAP-1 with an anti-Aspn/PLAP-1 antibody arrested the growth of the secondary follicles; 70% of them showed an abnormal shrunk morphology (Figure 5). Although Aspn/PLAP-1 has been shown to be involved in various biological and pathogenic processes including periodontal ligament mineralization and osteoarthritis (37–42), we originally provide evidence for a novel biological role of Aspn/PLAP1 as a key factor for the normal growth of the secondary follicles. It is also noteworthy that the growth of the secondary follicles is not regulated by gonadotropins and that its rigorous mechanisms has been poorly investigated, compared with gonadotropin-dependent follicle maturation and ovulation of antral stage onward (1–6, 8). In other words, combined with the specific expression of the Aspn/PLAP-1 gene in the outer layer of theca cells, we have also elucidated a novel endocrine/paracrine role of the outer layer of theca cells in gonadotropin-independent growth of secondary follicles (Figure 5), and also presume that theca cells are categorized as various subpopulations on the basis of the specific gene expression and their biological functions.

Aspn/PLAP-1 has been shown to bind to only TGF-β and BMP2 among the TGF-β superfamily including activins and Growth/differentiation factors, and to down-regulate TGF-β-Smad2/3 signaling cascade via binding to TGF-β (37–42). Moreover, blockade of Aspn/PLAP-1 triggered activation of this signaling cascade (Figure 6), leading to disruption of the growth of secondary follicles (Figure 5). Altogether, these results verified that Aspn/PLAP-1 participates in the regulation of normal growth of secondary follicles via inactivation of TGF-β-Smad2/3 signaling cascades (Figure 6). In other words, TGF-β signaling cascades are negative factors of the growth of secondary follicles. The involvement of TGF-β in proliferation of granulosa cells and theca cells and LH-induced androgen production has been extensively documented (1–3, 9–12). However, such studies have been limited to theca cells from antral follicles. Consequently, biological roles of TGF-β in secondary follicles, which are LH-insensitive, have yet to be investigated. Moreover, the functional correlation of these factors with antral follicle maturation has been controversial. This is due to variable distribution of gene and protein expression of TGF-β and their receptors among animal species and/or developmental stages of follicles (1–3, 9–12). For instance, the expression of the TGF-β gene was detected in granulosa cells and theca cells of preantral follicles in human and mice, but only in theca cells in pig and bovine, and these expression levels are temporarily altered (10, 11). Likewise, TGF-β was shown to suppress LH-induced androgen production in human theca cells, but not in their mouse counterparts (1, 2, 11). Such confounding findings regarding the effects of TGF-β may result from administration of TGF-β to cultured ovaries, given that the ovary includes various developmental stage-follicles, and thus, TGF-β may exhibit differential activities on follicles at the respective developmental stages. For instance, the effect of administrated TGF-β may over-exhibit, conflict, or suppress those of endogenous TGF-β. In keeping with these findings, studies using isolated theca cells may show biological functions from those residing in follicles, leading to the misunderstanding of authentic endogenous roles of TGF-β in theca cells. In this context, the specific localization of Aspn/PLAP-1 to the outer layer of theca cells (Figure 4B) and our culture system using isolated secondary follicles (Figure 5) eliminate these possible artifacts, leading to the elucidation of a novel biological role of Aspn/PLAP-1 in regulation of the normal growth of secondary follicles via suppression of TGF-β-Smad2/3 signaling cascades. In addition, Aspn/PLAP-1 was found to be expressed in the outer layer of theca cells and the adjacent interstitial cells of preantral and antral follicles as well as those of secondary follicles (Figure 4B). These results suggest that biological functions of Aspn/PLAP-1, namely those of TGF-β, in preantral and antral follicles are different from those in secondary follicles. Investigation of the Aspn/PLAP-1 regulatory mechanism in the preantral and antral development and maturation is underway.

In conclusion, the present study has provided fundamental gene expression profiles in theca/interstitial cells in mice, identified a novel theca/interstitial cell marker, and explored the novel biological role of Aspn/PLAP-1 in the growth of mouse secondary follicles at the gonadotropin-independent developmental stages.

## GenBank Accession Numbers

*Col1a2* (NM_007743), *Col3a1* (NM_009930), *Lum* (NM_008524), *Eln* (NM_007925), *Nid1* (NM_010917), *Fn1* (NM_010233), *Ccl2* (NM_011333), *Ccl21a* (NM_011124), *Cxcl12* (NM_021704), *Thy1* (NM_009382), *Calcrl* (NM_018782), *Pdgfra* (NM_011058), *Aqp1* (NM_007472), *Aspn/Plap-1* (NM_025711), *Cdh5* (NM_009868), *Cyp17a1* (NM_007809), *Bmp4* (NM_007554), *Bmp7* (NM_007557).

## Data Availability

The datasets generated for this study can be found in the Sequence Read Archive.

## Author Contributions

HS designed the research. MA, AS, SM, KH, TO, TK, KY, and HS performed the research. MA, AS, SM, TO, KY, and HS analyzed the data. MA, AS, TO, and HS wrote the paper.

### Conflict of Interest Statement

The authors declare that the research was conducted in the absence of any commercial or financial relationships that could be construed as a potential conflict of interest.

## Abbreviations

Aspn: Asporin
BMP: bone morphogenetic protein
Nid1: Nidogen 1
PLAP-1: periodontal ligament-associated protein 1
TGF-β: transforming growth factor-β.
